# Translating Molecular Technologies into Routine Newborn Screening Practice

**DOI:** 10.3390/ijns6040080

**Published:** 2020-10-15

**Authors:** Sarah M. Furnier, Maureen S. Durkin, Mei W. Baker

**Affiliations:** 1 Department of Population Health Sciences, University of Wisconsin School of Medicine and Public Health, Madison, WI 53705, USA; furnier@wisc.edu (S.M.F.); maureen.durkin@wisc.edu (M.S.D.); 2 Waisman Center, University of Wisconsin-Madison, Madison, WI 53705, USA; 3 Department of Pediatrics, University of Wisconsin School of Medicine and Public Health, Madison, WI 53705, USA; 4 Wisconsin State Laboratory of Hygiene, University of Wisconsin School of Medicine and Public Health, Madison, WI 53706, USA

**Keywords:** newborn screening, next generation sequencing, droplet digital polymerase chain reaction, real-time polymerase chain reaction, Tetra-primer amplification refractory mutation system–polymerase chain reaction, severe combined immunodeficiency, spinal muscular atrophy, cystic fibrosis

## Abstract

As biotechnologies advance and better treatment regimens emerge, there is a trend toward applying more advanced technologies and adding more conditions to the newborn screening (NBS) panel. In the current Recommended Uniform Screening Panel (RUSP), all conditions but one, congenital hypothyroidism, have well-defined genes and inheritance patterns, so it is beneficial to incorporate molecular testing in NBS when it is necessary and appropriate. Indeed, the applications of molecular technologies have taken NBS to previously uncharted territory. In this paper, based on our own program experience and what has been reported in the literature, we describe current practices regarding the applications of molecular technologies in routine NBS practice in the era of genomic and precision medicine.

## 1. Introduction

The goal of newborn screening (NBS) is to identify presymptomatic newborns with serious or fatal disorders that can be successfully treated, thereby achieving a significant reduction in morbidity and mortality. The more than 55-year history of NBS demonstrates that NBS is an extremely successful and cost-efficient public health undertaking in the field of preventive medicine. In the United States alone, approximately four million infants are screened annually, and over 12,000 are identified with one of the screened conditions and receive early medical attention [1].

As biotechnologies advance and better treatment regimens emerge, there is a trend toward applying more advanced technologies in the NBS context and adding more conditions to the NBS panel. In the current Recommended Uniform Screening Panel (RUSP), a list of conditions recommended for inclusion in state NBS programs by the Department of Health and Human Services, all conditions but one, congenital hypothyroidism, have well-defined genes and inheritance patterns, so it is beneficial to incorporate molecular testing in NBS when it is necessary and appropriate. Indeed, the application of molecular technologies has taken NBS to previously uncharted territory. In a 1989 publication, Jinks and colleagues reported a method to isolate DNA from NBS dried blood specimens and to use the isolated DNA to detect a pathogenic variant of the *HBB* gene that causes sickle cell disease [2]. In 1991, Wisconsin researchers adapted this DNA isolation method to detect *CFTR* c.1521_1523delCTT (also-known-as F508del), the most common variant associated with cystic fibrosis (CF), in a randomized clinical trial for NBS for CF in which NBS specimens with elevated immunoreactive trypsinogen (IRT), a biomarker elevated in newborns with CF, underwent F508del analysis [3]. This so-called IRT/DNA strategy in NBS for CF was later carried forward when routine NBS for CF was implemented in Wisconsin in 1994 [4]. The IRT/DNA protocol has also successfully been implemented in routine screening in NBS programs in Australia and Europe [5,6].

In recent years, more and more DNA-based assays have been integrated into routine NBS testing. In this paper, based on our own Wisconsin program experience and what has been reported in the literature, we attempt to present the current practice regarding molecular technologies in routine NBS practice in the era of genomic and precision medicine. Here we describe those applications in three categories (Table 1).

## 2. Molecular Marker Identification as Primary Screening Methods in NBS for Severe Combined Immunodeficiency (SCID) and Spinal Muscular Atrophy (SMA)

SCID is a disorder of abnormal development of the immune system, leading to an inability to mount an adequate immune response. When undiagnosed and untreated, the disorder has a high mortality rate, and the success of intervention is dependent on how early it is implemented [7]. Prior to the development of an NBS method for detecting SCID, at a 2004 Centers for Disease Control and Preventions conference, SCID was recognized as a disorder that met important criteria for inclusion in NBS [8]. Soon afterwards, Chan and Puck demonstrated that measurement of T-cell receptor excision circles (TRECs) in dried blood samples can be used for SCID screening in newborns [9] based on a method to study the effectiveness of anti-retroviral treatments in patients with acquired immunodeficiency syndrome by measuring TREC copies as a proxy for newly generated T cells after treatment [10]. Baker and colleagues adopted the concept of using TRECs as a SCID screening marker and further optimized the process of isolating DNA from routine NBS dried blood specimens in order to maximize DNA yield and improved the DNA amplification in a real-time PCR analysis. By doing so, they reduced the false positive rate [11].

In 2008, the Wisconsin NBS program implemented a molecular screening assay for SCID, the first DNA-based assay used as a first-tier screen in NBS history [11,12]. In 2010, SCID screening was added to the RUSP [13]. By the end of 2018, all NBS programs in the United States had added SCID to their NBS panels, and the experience of NBS for SCID in 11 screening programs throughout the United States was reported in 2014 [14,15]. Progress in population-based NBS for SCID has also been made in many other countries, including Saudi Arabia, Spain, France, Sweden, the Netherlands, Taiwan, Brazil, Japan, and Israel [16,17,18,19,20,21,22,23,24].

Although SCID screening methods are DNA-based assays, TRECs are not a genetic marker and are used in SCID screening as a surrogate marker of T-cell development [25]. Currently, there are two predominant methods for measuring TRECs in routine NBS for SCID: real-time PCR and end-point PCR [26]. Three reports in the literature have reported SCID screening methods that involve a combination of TREC assays and genetic testing by next generation sequencing (NGS). In addition to an end-point-PCR TREC analysis (EnLite Neonatal TREC Kit, Perkin Elmer, Turku, Finland), Al-Mousa and colleagues performed targeted NGS using a panel of genes known to be associated with primary immunodeficiencies (PIDs), later confirmed through Sanger sequencing. They also used a copy number-based assay (RT-qPCR and single nucleotide polymorphism microarrays) to detect the chromosome 22q11.2 deletion, a non-SCID PID [16]. Muramatsu and colleagues used a similar approach, but the chromosome 22q11.2 deletion was included in the NGS panel [23]. Strand and colleagues reported a two-tier approach in a pilot NBS for SCID: the first tier consists of real-time PCR for TREC and the second-tier NGS of a targeted panel of PID-associated genes with results confirmed by Sanger sequencing [27]. This combination method allows the addition of relevant gene variant information to infants identified by TREC assays as at high risk for SCID as a part of the routine NBS process which has been demonstrated by the above cited three reports.

SMA is an autosomal recessive neuromuscular disease caused by pathogenic variants in the survival motor neuron 1 (*SMN1*) gene. SMA is characterized by loss of lower motor neurons, or anterior horn cells, in the spinal cord and brain stem nuclei leading to progressive symmetrical muscle weakness and atrophy. It is the most common inherited cause of childhood mortality and, in the general population, the incidence is approximately 1 in 11,000 live births [28]. The survival motor neuron 2 (*SMN2*) copy number is a major modifier of the SMA phenotype with higher *SMN2* copy numbers associated with later onset and a milder phenotype. Ninety-five percent of SMA patients have a homozygous exon 7 deletion of *SMN1*, and infants with this deletion and two copies of *SMN2* account for the most common severe infantile form of SMA, Type I. However, symptom onset and disease severity in infants and children who have three or more copies of *SMN2* are more variable [29]. Based on a natural history study, the SMA mortality rate is 68% by the age of two years old [30]. Recent Food and Drug Administration-approved effective gene modulation and gene therapy treatments have enhanced the justification for adding SMA to NBS programs. In 2018, the Advisory Committee on Heritable Disorders in Newborns and Children recommended expanding the RUSP to include SMA caused by homozygous deletion of exon 7 in *SMN1*, and the Secretary of Health and Human Services accepted the recommendation [31]. Subsequently, the Wisconsin Department of Health Services added SMA to the Wisconsin NBS condition panel by an emergency rule, effective on 15 October 2019.

Most NBS programs that have implemented NBS for SMA use a real-time PCR assay to simultaneously screen for SCID and SMA. The PCR assay identifies the absence of exon 7 in the *SMN1* gene while simultaneously evaluating TREC copy numbers; the additional cost for an SMA screening test is minimal because it is multiplexed with an existing screening assay used to identify infants with SCID. In some programs, the *SMN2* copy number assessment is also carried out on specimens with no functional copies of *SMN1* to provide “just-in-time” supplemental information to clinicians. In Wisconsin, we use our laboratory-developed and validated triplex real-time PCR assay to simultaneously screen for SCID and SMA. In addition to TRECs and *SMN1*, the amplification of a reference gene, *RPP30*, is included in the assay as a quality/quantity indicator for DNA isolated from 3.2-mm dried blood NBS specimens. Specimens with absent *SMN1* amplification undergo a droplet digital PCR assay to assess copy numbers of *SMN2*. Infants with an absent *SMN1* result are reported as screening positive for SMA, with *SMN2* copy number information provided to the follow-up pediatric neurologists. With the *SMN2* copy number information, clinicians are better equipped to let families know what to expect and convey treatment options during their initial conversations with families following positive SMA screening result. At the first follow-up clinical visit, the physician orders a confirmatory *SMN1* and *SMN2* test through a diagnosis reference laboratory.

There are several publications regarding the experience of NBS for SMA [32,33,34,35,36]. We have summarized these experiences in Table 2. Kay and colleagues acknowledged the first year of the New York state SMA NBS resulted in a much lower SMA birth incidence rate than ones reported in the literature and offered some potential explanations [35].

## 3. Targeted Gene Variant Panel as Second-Tier Testing

As described earlier, the IRT/DNA strategy in NBS for CF started with one single *CFTR* variant, F508del. Yet as of 2010, many newborns in Europe and most babies in North America, Australia, and New Zealand are being screened with an IRT/DNA algorithm that often employs a *CFTR* variant panel with multiple CF-causing variants [4,6,37,38]. There has been a trend in recent years to add more CF-causing variants for screening. In 2015, a reported study demonstrated the feasibility of incorporating NGS into CF screening, which allows the inclusion of hundreds of *CFTR* pathogenic variants [39].

**Figure 1:**
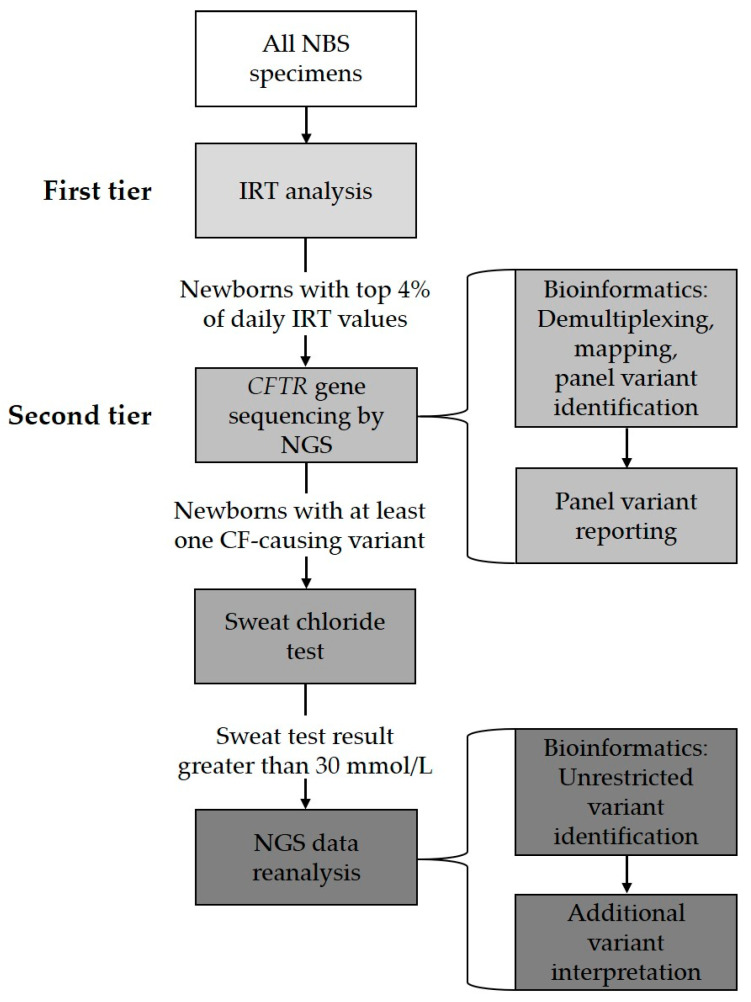
Wisconsin NBS for cystic fibrosis workflow.

Since April 2016, the Wisconsin NBS program has implemented such an NGS assay to identify CF-causing variants. Currently, IRT testing is the first-tier NBS test for CF. Specimens in the daily highest four percent of IRT values then undergo a second-tier *CFTR* variant analysis using NGS technology. A MiSeqDx Cystic Fibrosis 139-Variant Assay (Illumina, San Diego, CA, USA) is utilized to generate sequences of the *CFTR* gene coding regions and intron–exon junctions. These data are then filtered to display CF-causing variants. The assay-accompanied software identifies 139 CF-causing variants, including the most common *CFTR* deletion of exon 2 and exon 3 and *CFTR* deletion of exon 22 and exon 23. A laboratory-developed variant-call pipeline is also used to include additional CF-causing variants based on CFTR2, a website that provides information about whether the variant or variant combination is CF-causing and some functional analysis results with this variant or variant combination [40]. The current predefined panel consists of 324 CF-causing *CFTR* variants. Any newborn with one or more CF-causing variants identified is referred for a follow-up with a sweat chloride test as confirmatory testing. NGS data are reanalyzed for infants with sweat chloride greater than 30 mmol/L and one CF-causing variant identified through a regular NBS screening protocol. The NGS data reanalysis is performed by removing preset panel limitations and viewing all variants. This practice allows the identification of the additional CF-causing variants that are not included in the predefined panel and to identify infants with the *CFTR*-related metabolic syndrome, also known as Cystic Fibrosis Screen Positive, Inconclusive Diagnosis, which requires medical attention [41]. Figure 1 illustrates the Wisconsin CF screening process.

In the literature, several NBS programs have proposed using NGS to survey the whole *CFTR* gene as third-tier testing in CF NBS screening after the first-tier IRT analysis and second tier of single F508del analysis or a panel of *CFTR* variants for the purpose of maximizing the opportunity to identify two CF-causing variants in true CF screening positive cases [42,43]. A drawback of this approach is that it also identifies *CFTR* variants with unknown clinical significance.

## 4. Targeted Gene Variant or Variants as “Just-In-Time” Information

The third group of applications of molecular technologies in routine NBS is a targeted pathogenic gene variant analysis performed as a supplemental test after the biochemical or enzymatic test is completed and screening positive specimens have been determined. The “just-in-time” gene variant information is intended to help clinicians better interpret the biochemical or enzymatic testing results and be better prepared for their initial communication and discussion with families regarding NBS-positive results. Here we provide some examples from our NBS program to demonstrate the rationale and clinical utility of this practice.

For example, “just-in-time” information can help distinguish between different subtypes of a disorder. In the Wisconsin NBS program, galactose-1-phosphate uridylyltransferase (GALT) enzyme activity is the primary marker for galactosemia screening. Specimens with low or absent GALT activity undergo a *GALT* gene panel analysis. DNA isolated from dried blood specimens undergoes PCR amplification. With the Tetra-primer ARMS–PCR, the internal control, wild-type allele, and variant allele can be amplified simultaneously in the PCR reaction and, because of the difference in amplicon length, can be distinguished by agarose gel electrophoresis [44]. The variant panel includes *GALT* c.563A>G (Gln188Arg), c.404C>T (Ser135Leu), and c.940A>G (Asn314Asp). The *GALT* c.563A>G homozygote is the most common genotype in classic galactosemia. The *GALT* compound heterozygote of c.563A>G and c.404C>T causes clinical variant galactosemia, and the compound heterozygote of c.563A>G and c.940A>G results in Duarte variant galactosemia, which might not need clinical intervention [45].

For other disorders, “just-in-time” information can help identify the true disease status that otherwise would be masked by medical intervention prior to the collection of the dried blood spot, such as a blood transfusion. The primary screening method for sickle cell disease is a hemoglobin fraction pattern on an isoelectric focusing gel. However, this pattern could be altered when an infant receives a blood transfusion, and the hemoglobin fraction pattern for a newborn with sickle cell disease could appear instead as a sickle cell disease carrier pattern. In the Wisconsin NBS program, specimens collected after blood transfusion that present the sickle cell disease carrier pattern are subject to sickle cell disease genetic testing. DNA isolated from dried blood specimens undergoes PCR amplification with a pair of primers flanking the *HBB* gene variant c.20A>T region, which can be detected by a targeted Sanger sequencing analysis. The homozygous *HBB* c.20A>T result indicates the presence of sickle cell disease.

“Just-in-time” information can also be used to quickly detect genetic markers of disease in high-risk groups when immediate intervention is critical. Although maple syrup urine disease (MSUD) is rare with an incidence of 1 in 197,714 live births in the general population based on NBS data, among North American Old Order Mennonites, severe (”classic”) MSUD affects as many as 1 in 400 births due to a founder variant of *BCKDHA* (c.1312T>A, p.Tyr438Asn) [46,47]. Classic MSUD can present metabolic encephalopathy and critical brain edema within the first 24 to 48 hours of life. In order to identify newborns with classic MSUD in the Wisconsin Mennonite communities as early as possible, a dried blood specimen is collected one hour after birth, the specimen is delivered to the NBS laboratory by a family-arranged driver, and the laboratory runs the *BCKDHA* c.1312T>A assay based on the Tetra-primer ARMS–PCR immediately upon receiving the specimen. The testing results can be obtained as early as 10 h after birth. Another NBS specimen is collected between 24 and 48 hours after birth for routine NBS, including screening for MSUD using primary markers of leucine and isoleucine by mass spectrometry.

Finally, in the Wisconsin Pompe NBS pilot project, acid alpha-glucosidase (GAA) enzyme activity in dried blood spots is used as a primary screening tool. Specimens with GAA activity lower than 10% of the daily median are deemed as screening positive and undergo a *GAA* gene variant analysis. DNA isolated from dried blood specimens undergoes PCR amplification with primers flanking all *GAA* gene coding areas, and the *GAA* variant is detected by a Sanger sequencing analysis with the *GAA* variant database managed by Erasmus MC University Medical Center serving as a main reference for the pathogenicity assessment [48]. From 14 July 2017 to 31 March 2019, a total of 108,862 infants were screened with 13 infants screening positive based on GAA enzyme activities. Two pathogenic *GAA* variants were identified among these 13 infants screening positive, indicating late-onset Pompe disease. Genetic variant information in NBS for Pompe disease can help to determine variant-phenotype and genotype–phenotype correlations [49]. When performing the whole gene sequencing, it is possible to encounter gene variants with unknown significance, which need to be described in the report.

## 5. Discussion

We have described current applications of molecular technologies in routine NBS practice with the intention of illustrating their uses and assay principles. The NBS conditions presented here are examples and by no means an exhaustive list. Furthermore, some routinely used NBS practices involving molecular techniques might not have been reported in the literature at the time of this review. Molecular technologies will likely continue to play an important role in future NBS conditions, such as first-tier real-time assays for congenital cytomegalovirus infection [50,51], and the CF NBS two-tiered testing approach might be applicable to NBS for Duchenne muscular dystrophy [52,53]. Additionally, it is probable there will be increased desire to incorporate more complicated molecular or genetic assays in routine NBS—for example, whole exome or genome sequencing. These developments call for further considerations and a discussion of the many implications of technological advances in NBS, including their clinical utility, feasibility, bioinformatics support, interpretation and reporting of results, unintended findings and consequences, turn-around time, cost-benefit and effectiveness, and implications for data storage.

## Figures and Tables

**Table 1:** Molecular technology applications in Wisconsin routine newborn screening.

Molecular Application	Example Condition	Molecular Marker	Technology
First-tier markers	Severe combined immunodeficiency	T-cell receptor excision circles	Real-time polymerase chain reaction (PCR)
	Spinal muscular atrophy	Homozygous *SMN1* exon 7 deletion	Real-time PCR
Second-tier markers	Cystic fibrosis	*CFTR* variants	Next generation sequencing
Supplemental “just-in-time” ^1^ information	Galactosemia	*GALT* c.563A>G, c.404C>T, and c.940A>G	Tetra-primer amplification refractory mutation system (Tetra-primer ARMS)–PCR
	Maple syrup urine disease	*BCKDHA* c.1325 T>A	Tetra-primer ARMS–PCR
	Sickle cell disease	*HBB* c.20 A>T	Sanger sequencing
	Pompe disease	*GAA* coding region and intron-exon junctions	Sanger sequencing
	Spinal muscular atrophy	*SMN2* copy number	Droplet digital PCR

^1^ Screening positive determination is based on the first-tier result, and “just-in-time” gene variant information is intended to help clinicians better interpret the first-tier testing results and be better prepared for their initial communication and discussion with families regarding newborn screening positive results.

**Table 2:** Selected spinal muscular atrophy newborn screening studies.

Reference	Region	Screening Method	*SMN2* Inclusion	Number of Newborns Screened	Reported Incidence in Sample	Study Type
Chien et al. 2017 33	Taiwan	Real-time PCR *SMN1* assay to detect homozygous exon 7 deletion; verified by droplet digital PCR assay	Droplet digital PCR assay to assess *SMN2* copy number	120,267	1 in 17,181	Pilot
Boemer et al. 2019 32	Belgium	Real-time PCR *SMN1* assay to detect homozygous exon 7 deletion	No	Not applicable	Not applicable	Pilot
Vill et al. 2019 36	Germany	Real-time PCR *SMN1* assay to detect homozygous exon 7 deletion; verified by multiplex ligation-dependent probe amplification (MLPA)	MLPA to assess *SMN2* copy number	165,525	1 in 7524	Pilot
Kariyawasam et al. 2020 34	Australia	Real-time PCR *SMN1* assay to detect homozygous exon 7 deletion	Droplet digital PCR assay to assess *SMN2* copy number	103,903	1 in 10,390	Pilot
Kay et al. 2020 35	New York	Real-time PCR *SMN1* assay to detect homozygous exon 7 deletion	Real time PCR assay to assess *SMN2* copy number	225,093	1 in 28,137	Routine
