# Correction: Furnier et al. Translating Molecular Technologies into Routine Newborn Screening Practice. *Int. J. Neonatal Screen*. 2020, *6*, 80

**DOI:** 10.3390/ijns7040066

**Published:** 2021-10-22

**Authors:** Sarah M. Furnier, Maureen S. Durkin, Mei W. Baker

**Affiliations:** 1 Department of Population Health Sciences, University of Wisconsin School of Medicine and Public Health, Madison, WI 53705, USA; furnier@wisc.edu (S.M.F.); maureen.durkin@wisc.edu (M.S.D.); 2 Waisman Center, University of Wisconsin-Madison, Madison, WI 53705, USA; 3 Department of Pediatrics, University of Wisconsin School of Medicine and Public Health, Madison, WI 53705, USA; 4 Wisconsin State Laboratory of Hygiene, University of Wisconsin School of Medicine and Public Health, Madison, WI 53706, USA

In the original article [1], there was a mistake in Table 2 as published. For reference Vill et al. 2019, the entry “1 in 7096” has been corrected to “1 in 7524”. Also for reference Kay et al. 2020, the entry “No” under “*SMN2* Inclusion” has been corrected to say “Real-time PCR assay to assess *SMN2* copy number”. The corrected Table 2 appears below. The authors apologize for any inconvenience caused and state that the scientific conclusions are unaffected. The original article has been updated.

## Figures and Tables

**Table 2:** Selected spinal muscular atrophy newborn screening studies.

Reference	Region	Screening Method	*SMN2* Inclusion	Number of Newborns Screened	Reported Incidence in Sample	Study Type
Chien et al. 2017 [33]	Taiwan	Real-time PCR *SMN1* assay to detect homozygous exon 7 deletion; verified by droplet digital PCR assay	Droplet digital PCR assay to assess *SMN2* copy number	120,267	1 in 17,181	Pilot
Boemer et al. 2019 [32]	Belgium	Real-time PCR *SMN1* assay to detect homozygous exon 7 deletion	No	Not applicable	Not applicable	Pilot
Vill et al. 2019 [36]	Germany	Real-time PCR *SMN1* assay to detect homozygous exon 7 deletion; verified by multiplex ligation-dependent probe amplification (MLPA)	MLPA to assess *SMN2* copy number	165,525	1 in 7524	Pilot
Kariyawasam et al. 2020 [34]	Australia	Real-time PCR *SMN1* assay to detect homozygous exon 7 deletion	Droplet digital PCR assay to assess *SMN2* copy number	103,903	1 in 10,390	Pilot
Kay et al. 2020 [35]	New York	Real-time PCR *SMN1* assay to detect homozygous exon 7 deletion	Real-time PCR assay to assess *SMN2* copy number	225,093	1 in 28,137	Routine
